# Population Genetic Inference With MIGRATE

**DOI:** 10.1002/cpbi.87

**Published:** 2019-10-24

**Authors:** Peter Beerli, Somayeh Mashayekhi, Marjan Sadeghi, Marzieh Khodaei, Kyle Shaw

**Affiliations:** ^1^ Department of Scientific Computing Florida State University Tallahassee Florida; ^2^ Department of Mathematics Kennesaw State University Marietta Georgia

**Keywords:** Bayesian inference, coalescent, divergence time, DNA, gene flow, MCMC, microsatellite, population genetics

## Abstract

Many evolutionary biologists collect genetic data from natural populations and then need to investigate the relationship among these populations to compare different biogeographic hypotheses. MIGRATE, a useful tool for exploring relationships between populations and comparing hypotheses, has existed since 1998. Throughout the years, it has steadily improved in both the quality of algorithms used and in the efficiency of carrying out those calculations, thus allowing for a larger number of loci to be evaluated. This efficiency has been enhanced, as MIGRATE has been developed to perform many of its calculations concurrently when running on a computer cluster. The program is based on the coalescence theory and uses Bayesian inference to estimate posterior probability densities of all the parameters of a user‐specified population model. Complex models, which include migration and colonization parameters, can be specified. These models can be evaluated using marginal likelihoods, thus allowing a user to compare the merits of different hypotheses. The three presented protocols will help novice users to develop sophisticated analysis techniques useful for their research projects. © 2019 The Authors.

**Basic Protocol 1**: First steps with MIGRATE

**Basic Protocol 2**: Population model specification

**Basic Protocol 3**: Prior distribution specification

**Basic Protocol 4**: Model selection

**Support Protocol 1**: Installing the program MIGRATE

**Support Protocol 2**: Installation of parallel MIGRATE

## INTRODUCTION

Population genetics is concerned with the interpretation of the observed genetic variability in nature. With recent advances in DNA sequencing technology, researchers are able to generate sequence data from many individuals within a sampling location and from different species. Researchers are interested in understanding how the observed variability came about, and have therefore developed many population genetics models that connect theoretical models with real‐world data. Methods in this field are concerned with the correlated nature of the data, for example, the fact that all living individual organisms are related to each other even though the most recent common ancestor for some will be millions or billions of generations in the past. Kingman (1982) developed a probabilistic framework that allows connecting this relationship structure with the mutation process that shapes the genetic variability. Computer programs have been developed that use these frameworks and allow researchers to evaluate population genetic models in the light of observed genetic data. This article discusses the software MIGRATE (available at http://popgen.sc.fsu.edu and https://peterbeerli.com/migrate).

The computer program MIGRATE originated in 1998 (Beerli, 1998; Beerli & Felsenstein, 1999) as a maximum‐likelihood program analyzing asymmetric gene flow patterns between two populations using coalescence theory (Kingman, 1982). Beerli and Felsenstein (2001) extended the program to multiple populations. In 2006, the main author of MIGRATE (Beerli, 2006) modified the program to favor Bayesian inference over maximum likelihood. This change allowed handling more complex problems more easily. Version 3 of the software still allows one to run maximum likelihood analyses (MLA), but Bayesian inference is preferred. Version 4 removed the ability to run MLA, because MLA leads to narrow support intervals (Wilson et al., 2000), and comparison of models is more complicated than using a Bayesian selection approach. MIGRATE works with several different types of genetic data, such as DNA sequences, linked or unlinked single nucleotide polymorphisms, microsatellite repeat data, and allozyme data. The program frames the population‐genetic parameters as compounds with the mutation rate, which is generally unknown. Usually, researchers are interested in population size, *N*_e_, immigration rate, *m*, or divergence times. These parameters are expressed in MIGRATE as the mutation‐scaled effective population size of population i, Θ_i_, which is x*N*_e_^(i)^μ, where x is a constant that depends on the ploidy level of the data (data from diploid individuals use x = 4; for details, consult the MIGRATE manual at http://www.peterbeerli.com/programs/migrate/distribution_4.x/migratedoc4.x.pdf); M_j to i_ is the mutation‐scaled immigration rate into population i from j; M is defined as the immigration rate m/μ.  It is important to note that in the standard population genetics literature we only consider immigration and not emigration, which is equivalent to death. The divergence time is often measured in generations, but since we estimate the parameters from sequence data, the divergence time is confounded with the mutation rate. MIGRATE estimates the mutation‐scaled divergence time, which is in units of generation, ×μ.  For more details of parameter definition, inspect the manual.

This article describes all steps necessary to evaluate population genetics parameters using sequence data. We will use an example of simulated sequence data from a total of 30 individuals that were ‘collected’ from three locations. A biologist, who may have collected similar data from real populations, might wish to estimate the number of individuals at each location, the amount of gene flow among the locations, whether these locations are part of a single panmictic population, or if there are alternative population structures. One could also ask whether some locations were colonized from others. To answer all these questions, we will need sequence data that vary among the populations. One could imagine that if all individuals had the same identical sequence, we would simply be looking at clones, and all investigations of structure would fail. If the data are tremendously variable, we might declare that every individual is unique and a different species; in this case, our investigation should perhaps be a phylogenetic analysis and not a population‐genetic analysis with MIGRATE. Although the boundary between species delineation and population‐genetic analysis is fluid, MIGRATE can be used to explore this boundary.

Basic Protocol 1 walks a user through the first encounter with MIGRATE. It will help a user understand how to start, and also improve analysis to get consistent answers from the software. Basic Protocol 2 describes how to set up, modify, and test alternative population models. The Basic Protocol 4 explains how to compare models using the models and outputs from Basic Protocols 1 and 2. Basic Protocol 3 describes how to change prior distributions. There are two support protocols: Support Protocol 1 discusses the installation of the software on desktop and laptop computers and Support Protocol 2 describes the installation of the parallel MIGRATE version for cluster machines.

## NECESSARY RESOURCES

### Hardware

The user will need an up‐to‐date Macintosh, Unix/Linux, or Windows computer with at least 5 MB of free disk space for the program and 10 GB disk space for data and output files. The MIGRATE program does not need vast amounts of RAM, except when extensive sequence datasets are used (see Troubleshooting). Most runs will only need about 1 to 10 MB RAM, and can thus be run on up‐to‐date laptops without problems. For many problems, however, the program will require many hours of runtime. In these cases, a desktop or computing cluster will be necessary for the analysis.

### Software

The user will need the MIGRATE program, installed as described in the Support Protocol 1.

Depending on the operating system, users can download binary executable files or the source code. In general, downloading the program binary will be easiest for MacOS and Windows users. Compiling the source code on Mac computers requires a compiler such as GCC (https://gcc.gnu.org) or CLANG (https://developer.apple.com/xcode/ or https://clang.llvm.org); for Windows computers the compilation of MIGRATE is complicated and not discussed here.

Optionally, the user may employ data‐conversion tools to generate a MIGRATE dataset from other multiple alignment formats, for example PGDSpider (http://www.cmpg.unibe.ch/software/PGDSpider/) or Formatomatic (https://formatomatic.sourceforge.io). Although these programs convert to the MIGRATE format, users will need to check and compare the resulting datafiles with the examples in the MIGRATE manual, because the converters may have used an outdated template to generate their conversion routines. Users who know Python or R can generate MIGRATE datafiles easily from any source by writing their own scripts.

### Files

Appropriately aligned sequence data using the data format of MIGRATE will need to be prepared. This format is described in the manual (https://peterbeerli.com/programs/migrate/distribution_4.x/migratedoc4.x.pdf).

## STRATEGIC PLANNING

For a quick analysis, no strategic planning will be needed, but if a user wants to compare various population models, then it will be helpful to plan the analysis accordingly. Planning becomes essential with large datasets because some program runs may take several days to complete. Therefore, it will be important to make sure that enough computer resources are available (see protocols).

Runtime for a single run depends on both the amount of data, and, more importantly, the complexity of the model. As a rule of thumb, a run with *n* loci will take about *n* times longer than a run with a single locus. A population model with a single population will run quickly, but a model with more than 10 populations and complex interaction is challenging to set up, and the run will not be quick. Results for such a model will be poor, without many informative loci (*n >* 10).

Production runs with MIGRATE will usually takes hours; therefore, laptops are often not the best choice on which to run the program, and it would be best to either have dedicated desktop computer or to run the program on a computer cluster. For desktop runs, it is helpful to make sure that the program can run without interruption, for example, using the *nohup* facility on UNIX systems (we give examples of this in the basic protocols). It is optimal to run MIGRATE on a computer cluster that uses a batch system, because MIGRATE can be compiled as a data‐parallel program (see Support Protocol 2). The parallel version of MIGRATE can effectively parallelize the *n* loci if there are enough computer cores available. The parallel distribution of intermediate data adds overhead time, but parallel runs with more than five loci will always be faster than single‐CPU runs.

When MIGRATE is run on large clusters, it will be helpful to first run the data using a very short analysis (see Basic Protocol 1) on a local computer or a laptop. This procedure helps confirm whether the data were read correctly and whether the specified model gives correct results in a reasonable amount of time.

MIGRATE is a UNIX‐style command‐line executable; it should not be started by double‐clicking the file icon on a graphical user interface, but should be started within a command‐line environment. Users unfamiliar with UNIX terminals should familiarize themselves using tutorials available on the internet, for example: https://molevol.mbl.edu/index.php/UNIX or http://www.ee.surrey.ac.uk/Teaching/Unix/.

Keep the software manual handy, because options and datafiles are described in detail. You can download it from https://peterbeerli.com/programs/migrate/distribution_4.x/migratedoc4.x.pdf.

## Basic Protocol 1 FIRST STEPS WITH MIGRATE

This protocol assumes that the user has installed the program using the Support Protocol 1.

The steps below represent the first encounter of a novice user with MIGRATE. They show the basic operations needed to open and run MIGRATE on a dataset. The protocol does not discuss how to modify population models. It uses default values for all parameters and options for a very first run; however, further refinement will help to improve the results. Population model options and run‐time options will be discussed in Basic Protocol 2.

### Necessary Files

#### The datafile

The input data are specified in a file named infile by default. Before running MIGRATE, make sure that the infile is in the same directory as the executable migrate‐n, or that the path to the executable is known.

The infile can contain different types of genetic data, such as *DNA sequences*, *linked or unlinked single nucleotide polymorphisms*, *microsatellite repeat data*, or *allozyme data*. The MIGRATE manual contains examples of each of these datatypes and format specifications. It is absolutely necessary to study the data section in the manual before proceeding to run the program with your own data. Several converters take data in different formats and convert them to the MIGRATE‐specific format. For example, PGD‐Spider (Lischer & Excoffier, 2012) allows changing to MIGRATE data format from a large selection of other formats. Building the datafiles from scratch or using a scripting language is usually simple.

#### Description of the tutorial data set

**Figure 1:**
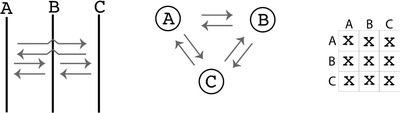
Default population model for three populations; all populations receive migrants from all others. Three different ways to represent the same population model are shown. The graph on the left represents each population in time, assuming gene flow (arrows) is recurrent among all of them; the middle graph represents only the interaction among populations without a time component; the matrix on the right is an adjacency matrix where the diagonal represents the population sizes and off‐diagonal represents immigration connections (more detail on this adjacency matrix in the Basic Protocol 2).

The dataset used for this protocol was simulated using a software application called *ms*, developed by Hudson (2002). This software generates a sample from a structured population. We simulated data for a sample of 30 individuals taken from three populations (see Fig. 1): two populations, named Arbon (A) and Berg (B), exchange a sizeable number of migrants (two migrants per generation); the third population split off from B and established a population in Chur (C). The populations A and B are equal in size and each is about one‐third of the population C. Sequence data were simulated for 10 independent loci of 1000 base pairs for each individual. We would expect that the data will make it difficult to establish that A and B are independent populations because they exchange more than one migrant per generation, but make it easy to recognize the divergence time between B and C. We set the population genetic parameters so that the data have sufficient variability to allow differentiation among some population genetic models.

### Parmfile

The parmfile is a text file that handles the program's runtime and model settings; it can replace the menu completely. It consists of six main sections:

#### General Options

Allows some MIGRATE‐specific options (these options should rarely be changed by users, but are described in the reference manual).

#### Data Options

Four main settings manage the different data types; *Infinite Allele*, *Stepwise Mutation*, and *Finite Sites Mutation* (this includes DNA/RNA sequences or single nucleotide polymorphisms), which are explained briefly in the parmfile.

#### Input Options

Other modifications of the input file, such as the file location, random number seed specification, and the title of the run can be set.

#### Output Options

This section defines the intermediate and final representation of results. The user can change the verbosity of the progress report, setting the filenames for the output, which will be written into a text file and a PDF file. The options also allow printing into a file of all visited genealogies during the run. There are several advanced options, such as recording the times of coalescent and migration events that will allow generation of skyline plots (see Drummond, Rambaut, Shapiro, & Pybus, 2005). The *Program output* contains detailed documentation about the individual parts of the output file.

#### Parameter start settings

MIGRATE uses a Markov chain Monte Carlo (MCMC) to generate the output. MCMC is a very general method to search a large, complex parameter space and record visited parameter values that are used to generate the posterior probability density distribution of all parameters of interest. To do this, we need to have start values for the parameters. These parameters can be drawn at random or can be set specifically.

#### Search strategies

MCMC methods run for arbitrarily long times; the runtime parameters define the length of the run and are important for the quality of the results. Runs that are too short will not explore the parameter space sufficiently; extremely long runs will deliver good results, but these results might also be achieved with much shorter runs. The defaults set for this section will lead to relatively short runs and almost always need improvement. This section defines the prior distributions needed for the Bayesian inference as well as the proposal distribution, and specifies the number of samples taken along the MCMC chain.

The resources for this basic protocol contain a datafile (infile), and three different parmfiles (parmfile_tooshort, parmfile_short, and parmfile_default). The run will take a few hours with the parmfile_default but only minutes with parmfile_tooshort. The runtime differences lead to different outcomes: the very short runs will not have converged, but this allows us to show how to improve the program run. These steps can then be used on the real data in similar ways.

### Installing the program

Follow the instructions in Support Protocol 1.

### Exploring the Menu of MIGRATE

In this protocol, we do not intend to get results from MIGRATE but to familiarize users with starting the executable and examine the menu options.

To run MIGRATE, you will need a specially formatted dataset which, by default, is named infile. The manual contains detailed instructions on the data format; also see the “Necessary Files” section. MIGRATE does not specifically recognize or ignore file extensions such as .txt; if you use these, they become part of the filename and cannot be omitted.

For most users, it may be easiest if the executable migrate‐n is located in the same directory as the datafile (see Support Protocol 1). On some systems that hide the file extensions, they should be made visible. We suggest that users on Macs check the box for “Show all filename extensions” or on Windows uncheck the box “Hide extensions for known file types.”

1 Start the program. The program can be run using one of the following commands in the command/terminal window:

**Table:** 

#if the system knows the path to executable
migrate‐n
#if the executable is in the same directory
./migrate‐n
#user specified location of executable
/pathtoexecutable/migrate‐n
  Once executed, MIGRATE displays a menu (Fig. 2). The header of the menu displays the type of the executable and its version number, and the current time and date. The main menu contains four different sub‐menus providing access to major sections of the program and two options. The first sub‐menu (D) permits manipulating data‐related parameters; with the sub‐menu (I), input and output‐related filenames can be changed; and (P) is a sub‐menu to manage the population models (see Basic Protocol 2). In this basic protocol, we discuss the crucial sub‐menu (S), Search Strategy; several options in this sub‐menu need to be changed to generate good results with the program. The (W) option saves all options into a file, by default named parmfile. This file is a text file and can be edited either with a text editor or through the menu. If you do not use this option, all changes will be lost once the program is closed.

**Figure 2:**
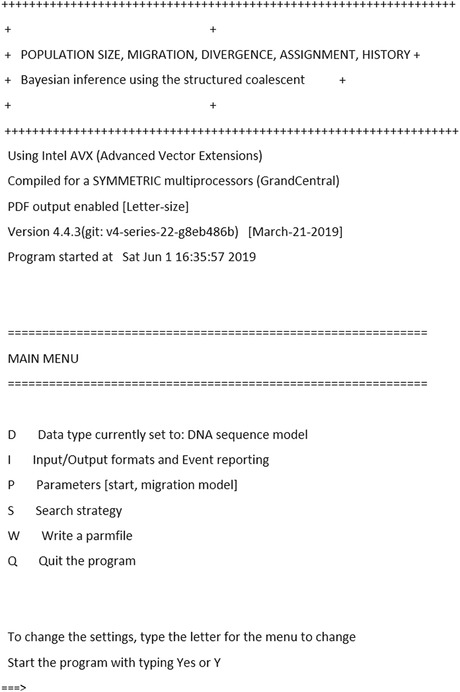
MIGRATE main menu.

2 Explore the submenus. Modify the search strategy by selecting (S), the Search Strategy menu, and then press 9 (see “9 Sampling increment?” in the menu) to change the sample recording increment. Use 3 for the increment. This change modifies the option *long‐inc* in the parmfile.
  MIGRATE does not record all the updates along the MCMC chain because that would lead to substantial intermediate storage requirements. The options **long‐sample** and **long‐inc**, under Bayesian MCMC Strategy method, are the number of sampled updates and the number of updates that are skipped, respectively. For example, with long‐inc=3 and long‐sample=6, MIGRATE will record parameter values for the steps 0, 4, 8, 12, 16, and 20.
3 Press Y to step out of the sub‐menu and go back to the main menu. Press W to save your changes into the file parmfile. Once you have done that, Press Q to quit the program.
  Note that we have not run any analysis yet, but have modified the parmfile.
4 Open the parmfile with a text editor (on the command line, use emacs or vi; graphical editors such a Textedit.app for Mac or Notepad for Windows will also work). Have a glance at the structure of the parmfile, compare with the description of the parmfile in Necessary Files, “Parmfile,” above. Search for **long‐inc** and verify that you changed that to 3 with the menu. Then, quit the file without saving.
  Options that start in the first column will be used; lines that start with # are comments and give explanations about the options.
  Once you have a parmfile written, you can rename the parmfile and use it. For example, you could rename the parmfile and run MIGRATE using the following commands:

**Table:** 

cp parmfile newparmfile

**Table:** 

migrate‐n newparmfile
  Then, MIGRATE will use the options from the newparmfile and not the defaults. We will see the use of custom parmfiles in the next section where we start with the actual tutorial.

### Running the program

This protocol aims to provide guidelines for novice users of MIGRATE. The first run applies a basic population genetic model to estimate mutation‐scaled population sizes and mutation‐scaled immigration rates from a dataset with three populations; therefore, there will be three population sizes and six immigration rates between all populations.

*Preparation*: Install the MIGRATE program using the instructions in Support Protocol 1. Then, download the file currentprotocols.tar.gz from https://peterbeerli.com/migrate/tutorials/ and unpack using these commands:

**Table:** 

curl ‐O https://peterbeerli.com/tutorials/currentprotocols.tar.gz tar zxvf currentprotocols.tar.gz cd currentprotocols/basic_protocol1

There is also a version of the tutorial deposited on github: https://github.com/pbeerli/currentprotocols.

Once the preparation step is completed, the user will have a directory basic_protocols1 containing the following files:

README
basic_protocol1.sh
basic_protocol1_mpi.sh
example_results
infile
parmfile_default
parmfile_short
parmfile_tooshort

The small dataset called infile will be used in this protocol; the other files will be discussed shortly. The infile dataset consists of 10 loci and three locations, named Arbon or A, Berg or B, and Chur or C. This dataset was simulated with a known population model and will be used for all protocols. Basic Protocol 1 treats all locations as populations with migration, and therefore does not need an adjustment of the custom migration model (which is explained thoroughly in Basic Protocol 2). Mastering the runtime specification (this protocol) and mastering model specification is important in order to achieve results with MIGRATE.

5 Create a directory and copy the infile and parmfile_tooshort into this directory; for example, in UNIX/Mac on the command line:

**Table:** 

# we assume that you are in the directory basic_protocol1 # this step creates a new directory, changes into it # and copies files to use mkdir temp_protocol1 cd temp_protocol1 cp ../infile cp ../parmfile_tooshort
6 Start the program. For instructional purposes, we use parmfile_tooshort. Now, run the command:

**Table:** 

migrate‐n parmfile_tooshort

If you run your own data, start migrate‐n without options or parmfile, then use the menu to change options and use the main menu option “(W) write a parmfile” to create a parmfile. The parmfile can be modified by hand or through the menu. Some options, in particular complex population models, are easier to edit using a text editor than using the menu. To use a hand‐modified parmfile, we suggest renaming the file, e.g., to parmfile_modified, and then calling MIGRATE‐n parmfile_modified. However, editing by hand has no fail‐safe mechanism and may lead to overwriting or even using the wrong options. Changes using the menu and then using the (W) option to rewrite the parmfile will always provide the correct option syntax.

**Figure 3:**
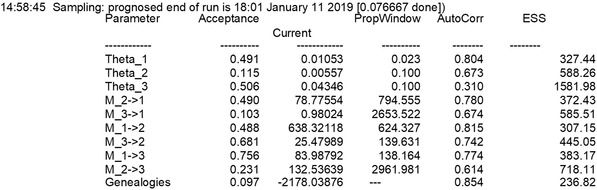
Example of an output during the run of the program: the information block contains a time stamp; the prognosed time of completion; parameter *acceptance* ratios; and *current* parameter value. The *Propwindow* column describes the size of the proposal window in the Markov chain Monte Carlo (MCMC) run, *AutoCorr* is the autocorrelation among parameter values, and *ESS* is the effective sample size of the MCMC.

7 For a first run, we use the options set by parmfile_tooshort without any additional changes. Once the menu is displayed, type Y or Yes to run the program. If the program cannot find your infile, it will show a warning, and you may be able to tell MIGRATE where your infile is. After three unsuccessful tries, it will quit. As soon as the program runs, it will create an output file called outfile in your directory.
  Usually, MIGRATE runs will take a considerable amount of time. The program defaults (parmfile_defaults) are set so that the example dataset runs for about 2 hr, but we are using parmfile_tooshort in this protocol first, and this will finish the run in a few minutes. For large datasets, even the default will lead to insufficient runtimes. The following steps will discuss how one can spot problems and improve the inference. During the run, the program provides information about the progress (Fig. 3).

**Figure 4:**
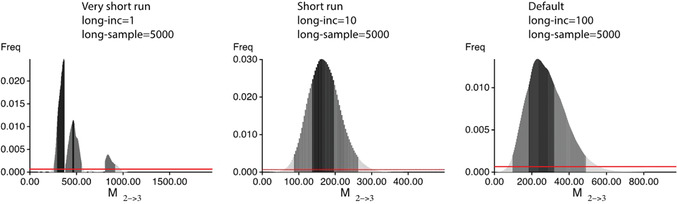
Example of histograms of the same mutation‐scaled immigration rates of three runs with different run length; the left run had set options that led to a runtime that is 100× shorter than the rightmost run. The leftmost histogram shows signs of problems.

8 Once the program has finished, check the content of outfile_tooshort and outfile_tooshort.pdf. outfile_tooshort will be created at the beginning of the run, and options will be copied into it for the first few seconds of the run. When the process finishes, outfile_tooshort will be filled with the results, and another file named outfile_tooshort.pdf will be created (the outfile_tooshort name is specified in the parmfile_tooshort that was used for this run).
  Both files contain the executed options and a table reporting the values of the posterior distribution of all parameters. The PDF file also includes histograms of these posterior distributions. If migrate‐n “finished” and did **not** create the PDF file, then it crashed! See Troubleshooting.
9 As a first step in the analysis of these files, you will need to figure out whether the run was appropriate:
  - Investigate outfile_tooshort.pdf
    This file contains a table with the mode, median, mean, and credibility sets of the posterior distribution of the parameters, and histograms depicting the posterior distribution for every estimated parameter. The parameters shown in the tables or histograms depend on the population model. If the values are all blank or zero, or very large, then most likely the run failed (see discussion of the Bayesian posterior probability table in Guidelines for Understanding Results; also see Fig. 4).
  - Inspect the histograms. If the histograms are jagged or show multiple peaks, this suggests that the run was not long enough, Figure 4 (leftmost) shows an example of a problematic posterior distribution. One can fix such problems by increasing the runtime, either by increasing the number of sampled steps (**long‐sample**) or by increasing the increment between the samples (**long‐inc**); the second option is less memory intensive and leads to results similar to the increase of the long‐sample option.
  - Additionally, you should inspect the *Effective Sample Size* (ESS) in the output file; if these numbers per parameter are not in the thousands, then there will likely be problems with the run. The ESS measures the number of independent samples taken throughout the program run; each step is dependent on (correlated with) the step before. The option **long‐inc** defines how many steps are not recorded to reduce this correlation. ESS should always be large; any number below 200 indicates that the runs need to improve.
  There are programs, such as the program **tracer** (Rambaut, Drummond, Xie, Baele, & Suchard, 2018), to evaluate the output (see in the Troubleshooting section for details on that), but often we do not need additional software to judge. The outfile.pdf is usually the best place for looking at the results.

10 Re‐run the analysis with more **long‐sample** or increased **long‐inc**: for example, use parmfile_short or parmfile_default and compare with the outputs from step 9. These parmfiles have increased **long‐inc**, but you can do this through the menu using “*(S) Search Strategy*” and then “*9 Sampling increment*.” MIGRATE will then show “How many steps (tree changes, parameter changes) to skip?”. Then, enter 10; this value of 10 is equivalent to the value in the parmfile_short. Then, type Y to step out of the menu to the master menu. Once at the master menu, press Y to run the program or use the command below:

**Table:** 

# we assume you are in directory temp_protocol1 cp ../parmfile_short parmfile_short migrate‐n parmfile_short ‐nomenu
  or for using long‐inc=100 use:

**Table:** 

# we assume you are in directory temp_protocol1 cp ../parmfile_default parmfile_default # for long runs on macs and unix, # we suggest to use the nohup facility nohup migrate‐n parmfile_default ‐nomenu > parm_default.log 2> parm_default.err
‐nomenu will start the program without directly going to the main menu. The nohup system command allows the continuation of the run even when the terminal window quits or the user logs out of the system; the output of the standard log to the screen will be captured in the file parm_default.log and errors will be captured in parm_default.err.
  Increase the runtime when the histograms do not look unimodal. In cases with low immigration rates, the histogram can peak at zero, or very close to zero, suggesting that the immigration parameter is close to zero. If the distribution peaks at the upper bound of the prior distribution, you will need to increase the prior distribution bound. For these changes, see Troubleshooting.
  With some datasets, it is very difficult to improve all the model parameters. With DNA sequence data, the population sizes are usually easy to estimate while immigration rates and divergence times are much more difficult. For microsatellite data, it is difficult to estimate population sizes reliably, but the immigration rates are less problematic. If the histograms of most of the parameters are unimodal, with a few that are not, and the model is complicated (see Basic Protocol 2), then one may need to stop and accept that some parameters cannot be well estimated with the data.
  In the tutorial package, there is also a file called basic_protocol1.sh that runs the complete sequence of Basic Protocol 1. It will take several hours:

**Table:** 

# we assume you are in directory basic_protocol1 . basic_protocol1.sh

## Basic Protocol 2 POPULATION MODEL SPECIFICATION

This protocol will help the user to create and change different population genetic models. MIGRATE can handle a variety of models that are specified through two options in the parmfile: an option that allows manipulating the connection among populations with an adjacency matrix (**custom‐migration**) and an option that manipulates the mapping of locations to populations (**population‐relabel**). The two options are explained in more detail below.

**Figure 5:**
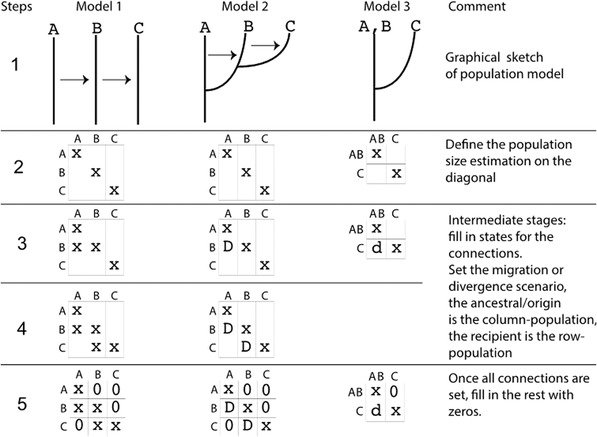
Example models. (1) Three models: recurrent immigration, recurrent immigration after divergence, and divergence (from left to right). (2) to (5) Suggested steps to build up the adjacency matrix. The populations used for the data and these models are named *Arbon* (A), *Berg* (B), and *Chur* (C). In the rightmost column, A and B were pooled and considered a single population AB.

Three different models are used in this tutorial. These models were chosen to demonstrate particular changes in the parmfile. More complex models are possible; the web page https://peterbeerli.com/migrate/tutorials.html will eventually contain more exotic examples. We will use the same dataset as in Basic Protocol 1, but will now adjust the population model specification in the parmfile. These specifications can be adjusted through the menu, but often it is easier to simply edit the parmfile with a text editor (make certain that your text editor is not tacking on invisible .txt file endings, because MIGRATE will need to know the complete filenames). The models are as follows; compare with Figure 5:

**Model 1**: Migrants from the population Arbon (A) arrive in Berg (B) and migrants from Berg arrive in Chur (C) (Fig. 5, column 1).

**Model 2**: Chur splits off Berg, and Berg splits of Arbon; after the splits, there is still a stream of migrants as in Model 1 (Fig. 5, column 2).

**Model 3**: Arbon and Berg are pooled into a combined population labeled “AB”; Chur splits off of “AB” with no migration after the split (Fig. 5, column 3).

Note that the **population‐relabel** option defines how the locations in the infile are used during the run. By default, every location is its own panmictic population. **population‐relabel** allows locations to be combined into populations by changing their labeling; the discussion of Model 3 in this protocol will describe this in detail.

The **custom‐migration** option is specified as a linearized adjacency matrix. Figure 5 introduces three different models and shows how the model graph can then be transformed into an option statement. The entries in the adjacency matrix define whether a population receives migrants from another population, if it is a population that split off from another ancestral population, or if it does not have contact with another population. Adjacency matrices must be connected graphs. An adjacency matrix with two disconnected sub‐graphs will lead to failure while running the program because two disconnected population groups can never coalesce when we reconstruct the relationships among individuals of the two groups.

### Necessary Files

Necessary files are the same as in Basic Protocol 1.

### Protocol Steps

We suggest reusing the parmfile that was used in Basic Protocol 1. Perhaps copy it from parmfile_default to parmfile_model1 for the first model and parmfile_model2 for the second, etc. Using parmfile_default will take considerable time; for a quick pass through this tutorial we suggest using parmfile_short, but that may lead to runs that are not consistent, and these will then lead to different model probabilities in the following Basic Protocol 4. You also may want to change the output file names before you proceed; this makes sure that you do not overwrite your earlier output files.

### Preparation

Prepare the *parmfile* for changing the population model. For a quick run through the tutorial use:

**Table:** 

# we assume you are in the directory currentprotocols cd basic_protocol2 mkdir temp_protocol2 cd temp_protocol2 cp ../../basic_protocol1/infile infile cp ../../basic_protocol1/parmfile_short parmfile_model1 cp ../../basic_protocol1/parmfile_short parmfile_model2 cp ../../basic_protocol1/parmfile_short parmfile_model3

for better results use:

**Table:** 

# we assume you are in the directory currentprotocols cd basic_protocol2 mkdir temp_protocol2 cd temp_protocol2 cp ../../basic_protocol1/infile infile cp ../../basic_protocol1/parmfile_default parmfile_model1 cp ../../basic_protocol1/parmfile_default parmfile_model2 cp ../../basic_protocol1/parmfile_default parmfile_model3

The next steps discuss the modification of the population model. This can be done in two ways—either through the menu or using a text editor. Designing new population models can be challenging. One approach is to (a) sketch the connection graph (examples are in Fig. 5, step 1) on a piece of paper; then (b) write out the adjacency matrix (or connection matrix) (Fig. 5, steps 2 to 5) among all populations; and then (c) fill in the options. We suggest using a text‐editor method because that simplifies building more complicated models from simpler models using copy/paste.

### Model 1

1a Draw the graphical model, label the populations, and copy the first model in Figure 5 (the first column from the left). Figure 5 contains all the information for the model, but it is important to learn to do this without guidance from the figure; for example, we can express the relationship among the three populations as a graph through time or at a particular time.
2a Draw the adjacency matrix—this is a square matrix where the diagonal elements mark the population sizes. If you label them with an ‘x’ or ‘*’, then this means that these population sizes will be estimated. Several other options are possible; you may want to explore them in the reference manual. All diagonal elements must contain a symbol other than 0 (see Fig. 5, row 2).
3a Fill in the adjacency matrix (steps 3 to 5 in Fig. 5). In your graph sketch, there is an arrow from A to B; this translates into a x or * in the first column and second row of the adjacency matrix. In general, we would say the FROM population are the column labels and the TO populations are the row labels.
  In your graph there is an arrow from B to C, draw an x in the column with the B and the row with the C; this has now filled the second column, third row.
  After the above changes, your sketch does not contain more information; fill all remaining elements of the matrix that you did not touch with 0.
  Now use your adjacency matrix and linearize it so that you concatenate the first row and the second row and the third row, for Model 1 this looks like: {x00 xx0 0×x}.
  Next, find in parmfile_model1 the line that **starts** with **custom‐migration** and then edit that line using your prepared linearized adjacency matrix to read:

**Table:** 

custom‐migration={x00 xx0 0×x}
  The {x00 xx0 0×x} indicates that the first population has no immigration, while the second population gets migrants from the first population, and the third population gets migrants from the second population. Thus, the three rows in step 5 of Figure 5, Model 1, have been converted to one row in the *custom‐migration* specification.
  The default for the custom‐migration option is custom‐migration={**}. For our Basic Protocol 1 example with three populations, this will be extended to custom‐migration={*********} (9 positions); remember * and x are equivalent, and spaces are inconsequential. MIGRATE will extend an incomplete option using the last character in the custom‐migration option.
  Next find in the parmfile_model1 the lines that start with **outfile** and **pdf‐outfile,** then edit these to the following and save your edits:

**Table:** 

outfile=outfile_model1 pdf‐outfile=outfile_model1.pdf
4a Run the program:

**Table:** 

migrate‐n parmfile_model1 ‐nomenu
  You can also use the **nohup** facility to run the models (see last example in the Basic Protocol 1).
  Look at the results and compare them to the Basic Protocol 1; there are fewer parameters in the tables and histograms. The same approach should be used with Model 2 and Model 3.

### Model 2

1b Draw the graphical model and label the populations, using Model 2 in Figure 5 as a guide.
2b Draw the square adjacency matrix. Mark the diagonal elements with x, indicating that that value will be estimated (see Fig. 5).
3b Fill in the adjacency matrix (steps 3 to 5 in Fig. 5). In the graph for Model 2, there is a divergence with recurrent immigration from the ancestral population ‘A’ to ‘B’; this translates into a **D** in the first column and second row of the adjacency matrix. We use the column label, here A, as the ancestor, and the row label, here B, as the descendent (for details refer to Figure 5 and also refer to the reference manual).
  In the graph, there is a divergence with recurrent immigration from the ancestral column population B to column C. This has now filled the second column, the third row of Model 2 with **D**.
  Your sketch does not contain any more information; fill all elements of the matrix that you did not touch with 0.
  Now use your adjacency matrix for Model 2 and concatenate the first row, the second row, and the third row. This looks like {x00 Dx0 0Dx}.
  The parmfile model2 needs to have this custom‐migration setting. Find the line that starts with **custom‐migration,** the edit the line so that it looks like:

**Table:** 

custom‐migration={x00 Dx0 0Dx}
  Then, find in the parmfile_model2 the lines that start with outfile and pdf‐outfile, then edit these to the following:

**Table:** 

outfile=outfile_model2 pdf‐outfile=outfile_model2.pdf
4b Run the program:

**Table:** 

migrate‐n parmfile_model2 ‐nomenu
  For details on the nomenclature for all the possible characters in the adjacency matrix, consult the manual. In Model 2, we used ‘D’, which specifies a divergence with consecutive immigration, whereas in the following Model 3, we will use ‘d’, which specifies a divergence without consecutive immigration.

### Model 3

1c Draw the graphical model and label the populations using Figure 5 (rightmost column) as a guide.
2c Draw the square adjacency matrix. Here, the adjacency matrix has changed from 3 × 3 into 2 × 2 because we pooled the first two populations as a combined population. Mark the diagonal elements with x, indicating they are to be estimated (see Fig. 5).
3c Fill in the adjacency matrix (steps 3 to 5 in Fig. 5). For Model 3, in the graph there is just a divergence from the ancestral column population ‘A, B’ to column ‘C’; this translates into a ‘**d**’ in the first column and second row of the adjacency matrix.
  Your sketch does not contain any more information; fill all elements of the matrix that you did not touch with 0.
  Use now your adjacency matrix for Model 3 and concatenate the first row and the second row. This looks like {x0 dx}.
  Adapt the custom‐migration setting in parmfile_model3 to:

**Table:** 

custom‐migration={x0 dx}
  We pooled the first two populations A and B; the parmfile_model3 needs thus a change of the **population‐relabel** option. Find the option in parmfile_model3 and then edit it so that looks like:

**Table:** 

population‐relabel={1 1 2}
  This option takes the ‘populations’ from the infile and pools them so that the first and second population are relabeled as population 1 and the third is relabeled as population 2.
  Then, edit in the parmfile_model3 lines that start with **outfile** and **pdf‐outfile** to read:

**Table:** 

outfile=outfile_model3 pdf‐outfile=outfile_model3.pdf
4c Run the program:

**Table:** 

migrate‐n parmfile_model3 ‐nomenu
  The example parmfiles and a script for Basic Protocol 2 can be found in the basic_protocol2 directory; Executing basic_protocol2.sh will execute all examples in a directory called temp_protocol2.

## Basic Protocol 3 PRIOR DISTRIBUTION SPECIFICATION

This protocol will help the user to specify the prior distributions.

Note that MIGRATE is a Bayesian inference program. In this framework, users will need to decide the probability distribution of the parameters that are used for the population model. This specification is usually done taking the data into account. For example, if we estimate the average height of humans and we know that no adult is smaller than 50 cm or larger than 300 cm, we could then use a prior that has bounds at these numbers and otherwise assume that the distribution is flat (a uniform distribution). The choice of distribution is arbitrary, and the defaults in MIGRATE are uniform distributions. For most parameters, the specification of the boundaries is not as simple as this.

### Necessary Files

We need two files: the infile, which was used in Basic Protocols 1 and 2, and parmfile_model3; also, for comparison, outfile_model3.pdf; these files were created in Basic Protocol 2 in the directory temp_protocol2.

1 Make a copy of the Model 3 parmfile:

**Table:** 

# we assume to be in the directory currentprotocols cd basic_protocol3 mkdir temp_protocol3 cp ../../basic_protocol1/infile. cp ../../basic_protocol2/temp_protocol2/parmfile_model3 parmfile_prior
2 We change the prior distribution for the population sizes using the menu in MIGRATE:

**Table:** 

migrate‐n parmfile_prior
  In the main menu, select *(S) Search Strategy*. Then, select and follow the *7 Prior distribution* submenu. Pick *1 Set Theta prior distribution?*, then choose **1** (the N option will be automatically filled from the datafile, but can be set here to 2 if you wish). To change to a different prior distribution, pick a number, e.g., pick 1; this will now set the exponential distribution. The next menu specifies bounds and mean for this distribution; set it to: 0.0, 0.06, and 0.1. Then, use “Y” to go back to the main menu, and press “W” to save the changes.
3 Changing the prior directly using a text editor. Open parmfile_prior using a text editor. Search for the section where the lines start with bayes‐priors; the lines should look like this after step 2:

**Table:** 

bayes‐priors= THETA * * EXPPRIOR: 0.00 0.06 0.1 bayes‐priors= MIG * * UNIFORMPRIOR: 0.00 5000.00 500.00 bayes‐priors= SPLIT * * UNIFORMPRIOR: 0.00 0.1 0.01 bayes‐priors= SPLITSTD * * UNIFORMPRIOR: 0.00 0.1 0.01
  The line with THETA shows the changes we did using the menu; for this protocol, we will only change the lines that contain SPLIT and SPLITSTD. These two lines change the prior distribution for the divergence parameters. After the changes the block should read:

**Table:** 

bayes‐priors= THETA * * EXPPRIOR: 0.00 0.06 0.1 bayes‐priors= MIG * * UNIFORMPRIOR: 0.00 5000.00 500.00 bayes‐priors= SPLIT * * EXPPRIOR: 0.00 0.08 0.1 bayes‐priors= SPLITSTD * * EXPPRIOR: 0.00 0.08 0.1
  The * * in the option specification stands for any parameter of the specific type. In principle, one can specify a prior for every parameter, but this option is not well tested and should not be used in current MIGRATE version.
4 Change all occurrences of outfile_model3 in the parmfile_prior to outfile_prior (there are two occurrences).
5 Now, run the program and compare the results with outfile_model3.pdf. The results will be similar but usually are shifted more to the left with exponential priors than with uniform priors.
  For the tutorial example, the results and the credibility intervals are similar; therefore, the prior influence is negligible. If the prior has a strong influence, then the data are probably not informative.

## Basic Protocol 4 MODEL SELECTION

MIGRATE is capable of helping you select an appropriate population model. Model selection is an important part of a population genetics analysis because using an inappropriate model will lead to inaccurate estimates of parameters. It is important to stress that there is no one right model. Rather, there is a spectrum of varying useful models. A user will be able to compare models to each other and compare the relative merit of each.

### Necessary Files

For this protocol, users will need to have completed Basic Protocols 1 and 2, and will need outfile_short or outfile_default and outfile_model1, outfile_model2, and outfile_model3.

1 In our example, we ran a total of four models (Basic Protocols 1 and 2). Our first run consisted of three separate populations with recurrent migration among all three populations. For Basic Protocol 2, we ran three additional models (Fig. 5). Copy the output files into a new directory:

**Table:** 

# we assume you are in the directory currentprotocols cd advanced_protocol mkdir temp_advanced cd temp_advanced cp ../../basic_protocol2/temp_protocol2/outfile_*. ls outfile*
  The command sequence should deliver the following files:
outfile_model1
outfile_model1.pdf
outfile_model2
outfile_model2.pdf
outfile_model3
outfile_model3.pdf
outfile_short
outfile_short.pdf
2 After MIGRATE completes a run, the results of the run are printed into an outfile. Inside the outfile, toward the end, locate the *Log‐marginal‐likelihood* table as shown in Figure 6 (we use ‘log’ as a shortcut for the natural logarithm [ln or log_e_]). There are three log marginal likelihood scores:. *Raw Thermodynamic*, *Bezier Approximated*, and *Harmonic Mean*. If the run was long enough (see Basic Protocol 1), then the *Raw Thermodynamic* score should be close to the *Bezier Approximated* score. The *Harmonic Mean* score is retained for historical reasons and should not be used. Use the *Bezier Approximated* score, because Beerli and Palczewski (2010) and Palczewski and Beerli (2014) showed that this approach delivers better approximations of the log‐marginal likelihood.
*Examine the outfiles*: The run with the parmfile_short of the Basic Protocol 1 led to the Bézier log marginal likelihood score in the “All” row of –23751.68. Basic Protocol 2 generated three more models: in our example run, they produce the marginal log‐likelihood scores of –23387.34, –23052.66, and –22170.83, respectively. We are interested in the model with the highest marginal likelihood; therefore, from these four models, we would choose Model 3 (–22170.83), as it produces the highest marginal likelihood of seeing the observed data.
  Your values will be not identical to those reported here; for comparison, we have included the output files used for this text in the directory advanced_protocol/comparison.

**Figure 6:**
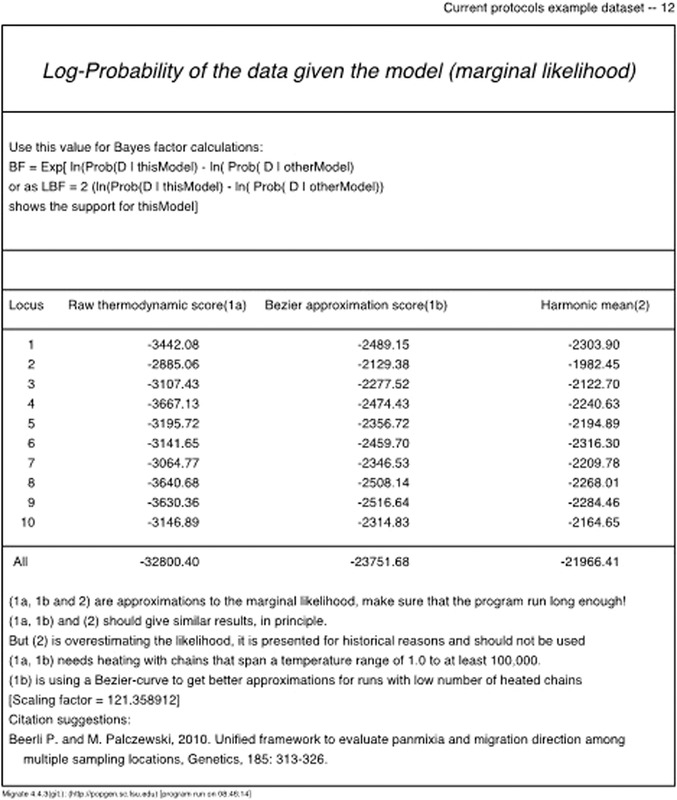
Copy of the Log marginal likelihood table as displayed in the outfile_short.pdf of the run that was carried out according to the instructions in Basic Protocol 1 using parmfile_short. A re‐run of the same data will lead to slightly different values.

3 We can quantify this result using a model probability approach (Burnham & Anderson, 2002). In this approach, we calculate the relative weight of a specific model compared to all analyzed models; we sum the marginal likelihoods of all models; and then calculate the model probability for each model scaled with this sum (Equation (1)):
(1) $$P\left(\mathrm{model}\right)=\frac{{\mathrm{mL}}_{\mathrm{model}}}{\sum_{i}{\mathrm{mL}}_{i}}$$
  where *mL* is the marginal likelihood.
  However, MIGRATE reports the log_e_ *mL*! The above calculation is complicated by the fact that the reported log marginal likelihood values are often large negative numbers, and a standard calculator will return zero. For example, exp(ln *mL*) = exp(–22170.83) will lead to 0.0 on most computer systems, and the sum cannot be calculated accurately. We can use a trick with a scalar *a*, where *a* is the same as the largest value among the log marginal likelihoods of all models, then use Equation 2:
(2) Pmodel= exp (lnmLmodel−a)∑i exp (lnmLi−a)
  In our example, we use the highest log marginal likelihood, set a=−22170.83, and then calculate P(model) for all models. This model probability makes it possible to discuss superiority of a particular model—here, Model 3 over the others. We can also calculate the log Bayes factor, which is the difference between the log marginal likelihoods of two models, for example, Model 2 compared with Model 1 is −23052.66−22170.83=−881.83; Bayes factors that are more than 10 units different are considered to be decisive. This suggests that the two models are different and that the model with more support (Model 3) wins over the other model (Model 2).
  Models must be run on the same data to be accurately compared. We have supplied a Python script (see the file bf.py in the tutorial package) that can do these calculations automatically. The script uses the line with Bézier log marginal likelihood and then calculates log Bayes factors, which are the differences of the log marginal likelihoods of the two models, and the model probability (Table 1). Looking at the values in Table 1, it becomes clear that the Model 3 is orders of magnitude better than the other tested models, and we should discuss this population model and its parameters in the report that discusses the data. The MIGRATE website will have more tutorials on how to handle such comparisons.

**Table 1:** Model Comparison of Four Models^a^

Model	Log(mL)	LBF	Model probability
Basic	−23751.68	−1580.85	0.00
Model 1	−23387.34	−1216.51	0.00
Model 2	−23052.66	−881.83	0.00
Model 3	−22170.83	0.00	1.00

a The model with the highest model probability is the best found model. The data were simulated from a model that had a very high immigration rate from A to B and a population split that separated C from A and B. This table was produced with the UNIX command grep “All “ outfile * | sort ‐n ‐k 4,4 | python bf.py.

4 Create the table with your own data (this will need a UNIX‐style command line):

**Table:** 

# we assume that you are in the directory currentprotocols cd advanced_protocols/temp_advanced grep “All “ outfile_* | sort ‐n ‐k 4,4 | python ../bf.py
  If this command fails, make sure that you have bf.py in the parental directory and a version of Python available; but steps 1 to 2 should give enough instructions to recreate the table by hand.
  This concludes Basic Protocol 4. The user may wonder why Model 3 was considered the best model. We simulated the sequence data for the three locations so that the locations Arbon and Berg have a high immigration rate between them (in fact, the gene flow is high enough that we may consider this a single population) and the location Chur was colonized later from Arbon/Berg; therefore, any of the three‐population models should explain the data less well than a two‐population model. We could test for many other models, but to be statistically consistent, users should define the models before they start testing, for example, by using existing biogeographic hypotheses to formulate models.

## Support Protocol 1 INSTALLING THE PROGRAM MIGRATE

Migrate is open‐source software and can be accessed at http://popgen.sc.fsu.edu or at http://peterbeerli.com/migrate. For this tutorial, we will need the MIGRATE‐4 series. The download section (https://peterbeerli.com/migrate/download_version4/) gives a list of packages to install. For Windows and MacOS, pick the appropriate binary package; for Linux, pick the src package (the source package can also be installed on MacOS if a compiler is available). For example, the following commands download the source distribution and will generate a directory migrate‐4.4.4 (or a newer version number):

**Table:** 

curl ‐O https://peterbeerli.com/migrate/d4/migrate‐newest.src.tar.gz tar zxvf migrate‐newest.src.tar.gz

Check the version number of the unpacked directory; for the summer of 2019, it is 4.4.4, but this will change on a regular basis. The migrate‐4.4.4 directory contains several directories and files. For a basic installation, the user will need to read the README textfile. The HISTORY textfile gives an overview over the software from the start of the distribution to today. The src directory contains all the source code, and the contribution directory contains helper programs that users have contributed (not discussed here). For standard installation, the compilation step for MIGRATE reduces to:

**Table:** 

# migrate source install procedure cd migrate‐4.4.4/src ./configure make sudo make install

The README gives more information. The distribution of Mac and Windows executables is simpler, because the user will only need to copy the files to the appropriate directories. For a Mac, the easiest procedure is to use the following command on the command line:

**Table:** 

# migrate binary install procedure cd migrate‐4.4.4 sudo mkdir ‐p/usr/local/bin sudo cp migrate‐n/usr/local/bin/

The user will need to be an administrator of the computer to use the sudo command that elevates the permissions from user to administrator, because /usr/local/ is usually protected. For Windows, the easiest way is to keep the migrate‐4.4.4 folder in your directory and add the path to the directory that contains the executable migrate‐n.exe to the system path file. For a session, you can change the path using:

**Table:** 

set path “%path%;C:\your\path\tomigrate”

For permanent solutions, you will need to use setx, but that may be tricky if the path is already long, because the path used in Windows has fixed maximal length. A safe solution is setting the path per session (using the command above), or putting the program into the same directory as the data and parmfile.

## Support Protocol 2 INSTALLATION OF PARALLEL MIGRATE

Installation of the parallel MIGRATE will need the source code and a compiler, and will also require additional software packages that handle the data‐parallel distribution, such as OPENMPI (https://www.open‐mpi.org) or MPICH (https://www.mpich.org). This support protocol will not address all installation steps of this additional package, but there are README files in the migrate directory and other help documentation on the internet and through the Google group *migrate‐support*. The following outline works on Macintosh operating systems and also on UNIX‐style operating systems, and installs OPENMPI into /usr/local; for a computer cluster, this directory would be best shared among different computers. MIGRATE is not strongly dependent on a fast transport protocol; therefore, fast ethernet connections among nodes in the computer‐cluster will work fine. Setting up the computer cluster is beyond the scope of this tutorial, but the README files in the OPENMPI package will help. The following instructions work well on a single computer with a large number of CPU cores.

1 Download and install OPENMPI:

**Table:** 

curl ‐O https://download.open‐mpi.org/release/open‐mpi/v4.0/openmpi‐4.0.1.tar.gz tar zxvf openmpi‐4.0.1.tar.gz cd openmpi‐4.0.1 ./configure make sudo make install
2 Compile the parallel MIGRATE. It is assumed that the MIGRATE source is already downloaded (see Support Protocol 1) and that the user has run ./configure and compiled the single‐CPU version:

**Table:** 

cd migrate‐4.4.4 ./configure make clean make mpis sudo cp migrate‐n‐mpi/usr/local/bin/
3 Verify that the parallel version works:

**Table:** 

cd migrate‐4.4.4/example mpirun ‐np 4 migrate‐n‐mpi parmfile.twoswisstowns
  The MIGRATE menu must display “Compiled for a PARALLEL COMPUTER ARCHITECTURE.” You can either run the program or quit (Q; or use control‐C because on some systems the Q option in the menu fails).
  Now, in all places where this tutorial suggests running *migrate‐n*, you can use mpirun ‐np X migrate‐n‐mpi. The X is the number of cores on your system. Migrate uses a master‐worker architecture that makes it possible to run different loci and replicates in parallel. The program uses its own load‐balancing system for the X cores. If you have X cores and the number of loci multiplied by replicates is smaller than X, then some of the worker nodes will remain idle; the best strategy is X = loci × replicates + 1.

## GUIDELINES FOR UNDERSTANDING RESULTS

The outfile.pdf contains all information after the run. An additional textfile, outfile, includes the same information except that it does not contain any figures; the text file can be used to electronically extract parts of tables, for example, the values for the marginal likelihoods used in the Basic Protocol 4. The output is organized in sections:

- *Header*: Contains the version number of MIGRATE and information about how the program was compiled, and also contains date and runtime information.
- *Options*: All options including the population model, priors, and run length are shown.
- *Bayesian posterior table*: This is a main output of the program. The table gives the mode, mean, median, and percentiles of the posterior probability density for each parameter.
- *Histograms*: The posterior distribution for each parameter is shown as a histogram. The histograms are color‐coded so that values that are in the interval of 25% to 75% credibility intervals are black, values in the 2.5% to 25% and 75% to 97.5% interval are gray, and values outside the 2.5% and 97.5% intervals are white. The red line marks the prior distribution.
- Marginal likelihood table for each locus and locus summary (see details in Basic Protocol 4).
- Runtime statistics, recordings of acceptance ratios, and effective sample sizes recorded during the run for each parameter.

### The Bayesian posterior probability table

**Figure 7:**
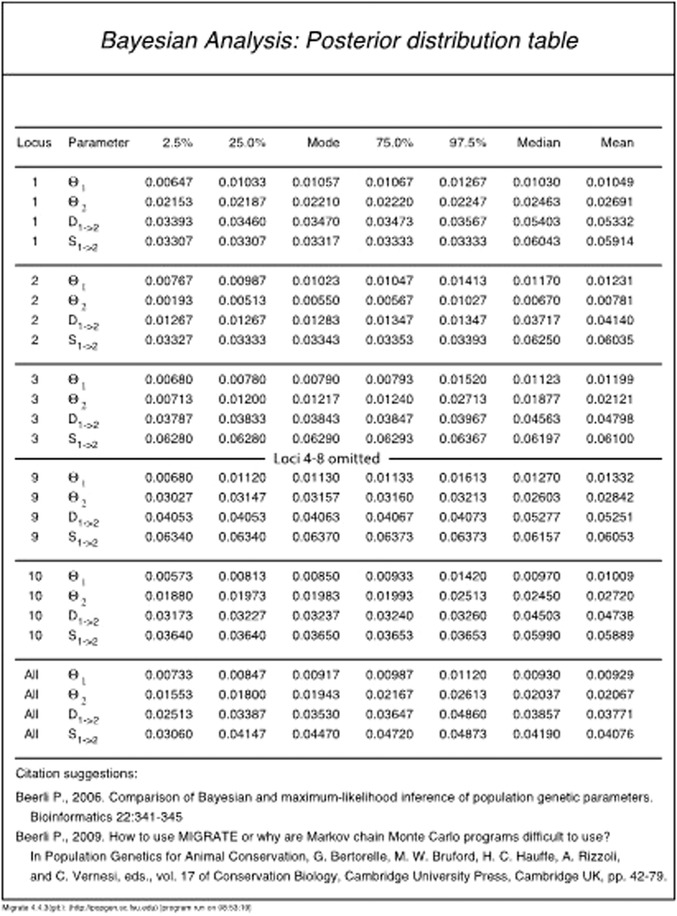
The main table in the output of MIGRATE. This table was produced with Model 3 in Basic Protocol 2. The full table is available in the downloaded tutorial material at currentprotocols/basic_protocol2/example_results/outfile_model3.pdf.

Here, we will discuss the Bayesian posterior probability table in detail for Model 3 of Basic Protocol 2 to give an idea what conclusions can be drawn from such an analysis. Figure 7 shows the results from Model 3 of Basic Protocol 2: the columns are the parameters, the 2.5% percentile, 25% percentile, mode, 75% percentile, 97.5% percentile, mean, and median for each locus, and a summary column over all loci. The summary over all loci is not a simple mean over all loci, but the product of all distributions of all loci. This table allows discussing the result of the analysis. For example, the mode of the mutation‐scaled population size of population 1 (combined locations Arbon and Berg) is Θ_1_ = 0.00917, and the mode of the population size of Chur is Θ_2_ = 0.01943. The size of Arbon/Berg is about 48% of Chur. The credibility interval for the size of Arbon/Berg is 0.00733 to 0.01120, and for Chur this is 0.01553 to 0.02613. The most extreme values for the size ratio are then 28% or 72%. The 95% credibility interval for the time of the colonization event that created the population Chur was 0.02413 to 0.04860, and its mode was at 0.03530. The units for the divergence time are in generations multiplied by expected mutations (gen*µ); we can express this scaled time in units of population size by dividing the scaled divergence time by the total population size (Θ_1_ + Θ_2_ = 4 *× N_e_* ^@^ *µ* where *N_e_* ^@^ is the combined population size of all populations). For example, the divergence event was 0.03530*/*(0.00917 + 0.01523) = 1.45 coalescence units in the past. In this case, the units are in terms of 4 *× N_e_* [we calculated gen*µ/(Θ_1_ + Θ_2_) = gen*/*(4 *N_e_*)]. We may wonder how accurate these estimates are. We simulated the data using the simulation program *ms* developed by Hudson (2002) with parameters so that populations and A and B combined had a size that was 67% of population C (we estimated 60% in our example!). Results for immigration and divergence will rarely be very precise and usually have a large credibility interval. We simulated the split of C from A and B at a time that was 2 *×* 4*N_e_* ^@^. Our estimated value of 1.45 underestimates somewhat, but this seems not to be uncommon with large divergence times. If we look at the divergence time using the credibility intervals, we can get an upper bound of the divergence time of 0.04860*/*(0.00733 + 0.01553) = 2.12, which would include the simulated divergence time.

### COMMENTARY

#### Background Information

Understanding the output of the program MIGRATE takes effort and time; the reference manual and tutorials on the MIGRATE website have additional information; another tutorial is available at http://peterbeerli.com/workshops/mbl/2018/tutorial/. Analyses of very large datasets will require considerable effort and time to complete. All options of MIGRATE are described in its reference manual (http://www.peterbeerli.com/programs/migrate/distribution_4.x/migratedoc4.x.pdf). In our view, the capability for model comparison makes MIGRATE an important tool for researchers with genetic data from natural populations.

Inferences of population genetic parameters from genetic data date back to work by Sewall Wright (Wright, 1951) developing estimators for his homozygosity index F and his work on F_ST_ (for a textbook on population genetics, use the small volume by Gillespie, 2004). The differences in the variabilities of allele frequencies at different locations allow the summary statistic F_ST_ to be used for describing the interaction among populations. F_ST_ measures the variability differences between subpopulations compared to the pooled total population. This is a very popular method for estimating relative magnitudes of gene flow among populations. However, it has shortcomings because F_ST_ cannot estimate asymmetric immigration rates or differentiate between population divergence and low immigration rates.

Coalescent estimators, such as MIGRATE, have no problem estimating complex models with asymmetric gene flow directly. The biggest drawback of full coalescent estimators is the length of runtime and the complexity of setting up the analysis.

In contrast to F_ST_‐based analyses, coalescent‐based methods, such as MIGRATE in particular, allow for setting up different hypotheses and then comparing them using a Bayes factor framework. Models are then compared to each other using the marginal likelihood. The marginal likelihood can be inferred by Bayesian inference.

In Bayesian inference, we calculate posterior probability densities of the parameters of a model. This posterior is proportional to the prior probability of the parameter multiplied by the probability of the data given the parameter of the model (likelihood of the parameter). When we integrate this quantity over all parameter values, we get the correct scaling of the posterior. This integral is the marginal likelihood. The marginal likelihood is the probability of seeing your data given your model.

MIGRATE estimates the marginal likelihood using thermodynamic integration (Beerli & Palczewski, 2010; Palczewski & Beerli, 2014).

MIGRATE allows users to estimate complex population models and also assess how well these models fit the data at hand. It is a complicated but versatile tool for practical population geneticists or conservation biologists.

### Troubleshooting

If users have difficulties with the program, the best way to resolve the problem is to ask questions on the MIGRATE support group (https://groups.google.com/forum/#!forum/MIGRATE‐support), but the following situations are common.

#### Program crashes immediately after start

The most common error with MIGRATE is a problem with the datafile. MIGRATE is very picky about the length of the names of the individuals in the dataset; the default is 10, which means there must be 10 characters or spaces for each individual name. The encoding of the file also needs to be ASCII. Errors in the datafile can be easily fixed by users, but sometimes these errors are difficult to detect in large datasets. A divide‐and‐conquer approach often helps. For example, cut the data in half and try to run, and then add data until it breaks again. For other errors, it may be best to consult the *MIGRATE‐support@gmail.com* mailing list, where the program author Peter Beerli or other MIGRATE users will give answers.

#### Problem with histograms

Sometimes the histograms in the PDF file do not display. There are two potential reasons. (1) Your Adobe Acrobat Reader fails to read the document. Because the program uses a PDF library to construct the histograms one after the other and positions these graphical objects onto the page, if you see that the first histogram displays well, and consecutive histograms only show the black axes without any labels, and/or blank pages, then you will need to use a different PDF viewer. We recommend Preview.app on MacOS or Nitro PDF viewer on Windows. (2) If the program only samples a few different states during the program run, then the histogram display can fail. The remedy for this would be to run the program longer (see Basic Protocol 1).

#### Problem with non‐convergence

In Basic Protocol 1, we outlined a scheme to improve the results by checking the histograms or using the Effective Sample Size (ESS) to judge whether the program may have converged on good answers or not. There are other tools, such as the program *tracer* (https://github.com/beast‐dev/tracer), to check for convergence. MIGRATE will need to set an additional option to generate a file that tracer can read. In the parmfile, set the option bayes‐allfile=NO to bayes‐allfile=YES:1:bayesallfile and then run MIGRATE. The manual discusses this file in more detail. This will now generate a bayesallfile that contains a detailed history of the MCMC run. The output can be read by tracer, but the bayesallfile can only contain a single locus; therefore, the MIGRATE distribution package contains a splitting tool to split the bayesallfile into files for each locus to help with this limitation, but currently there is no tool to summarize over loci. The best approach is the one outlined in Basic Protocol 1.

### More Advanced Options

Our outline of the protocols ignores the effect of mutation models on the results. Once users become familiar with the basic operations of MIGRATE, they should investigate the use of different mutation models, as MIGRATE does not co‐estimate mutation parameters. Usually, users should estimate mutation parameters employing a phylogenetic program such as PAUP* (http://paup.phylosolutions.com) to estimate the best mutation model. For example, the user should investigate whether the data will need a model that can take advantage of site‐rate variation.

Sometimes users have datasets with vast numbers of individuals or very uneven numbers of individuals; inferences using the coalescent do not need hundreds of individuals but need many independent loci. Datasets that have hundreds or thousands of individuals are difficult to analyze and will take a very long time to run. A better approach is to sub‐sample the dataset and run that; MIGRATE allows one to do this using the option random‐subset=number<:seed>, where number is the number of individuals in the population and seed is the random number seed to use to extract random individuals from the population. This is different from the general random number seed so that users can extract the same individuals to run different models. For model comparison, it will be imperative for the data tested for each model to be the same. More detail is available in the manual.

The program MIGRATE is actively maintained and improved; thus, it may be worthwhile to participate in the migrate‐support Google group or the Facebook page *@migratesoftware*.
